# An unbiased approach of molecular characterization of the endometrium: toward defining endometrial-based infertility

**DOI:** 10.1093/humrep/dead257

**Published:** 2023-12-14

**Authors:** Bich Ngoc Bui, Arif Ibrahim Ardisasmita, Ewart Kuijk, Signe Altmäe, Gaby Steba, Shari Mackens, Sabine Fuchs, Frank Broekmans, Edward Nieuwenhuis

**Affiliations:** Department of Gynaecology and Reproductive Medicine, University Medical Center Utrecht, Utrecht, The Netherlands; Department of Metabolic Diseases, University Medical Center Utrecht, Utrecht, The Netherlands; Department of Pediatric Gastroenterology, Wilhelmina Children’s Hospital, University Medical Center Utrecht, Utrecht, The Netherlands; Department of Biochemistry and Molecular Biology, Faculty of Sciences, University of Granada, Granada, Spain; Department of Gynaecology and Reproductive Medicine, University Medical Center Utrecht, Utrecht, The Netherlands; Brussels IVF, Universitair Ziekenhuis Brussel, Vrije Universiteit Brussel, Brussels, Belgium; Department of Metabolic Diseases, University Medical Center Utrecht, Utrecht, The Netherlands; Department of Gynaecology and Reproductive Medicine, University Medical Center Utrecht, Utrecht, The Netherlands; Centre for Infertility Care, Dijklander Ziekenhuis, Purmerend, The Netherlands; Department of Pediatric Gastroenterology, Wilhelmina Children’s Hospital, University Medical Center Utrecht, Utrecht, The Netherlands; Department of Science, University College Roosevelt, Middelburg, The Netherlands

**Keywords:** endometrium, molecular phenotyping, unexplained infertility, biological mechanism, single-cell analysis

## Abstract

Infertility is a complex condition affecting millions of couples worldwide. The current definition of infertility, based on clinical criteria, fails to account for the molecular and cellular changes that may occur during the development of infertility. Recent advancements in sequencing technology and single-cell analysis offer new opportunities to gain a deeper understanding of these changes. The endometrium has a potential role in infertility and has been extensively studied to identify gene expression profiles associated with (impaired) endometrial receptivity. However, limited overlap among studies hampers the identification of relevant downstream pathways that could play a role in the development of endometrial-related infertility. To address these challenges, we propose sequencing the endometrial transcriptome of healthy and infertile women at the single-cell level to consistently identify molecular signatures. Establishing consensus on physiological patterns in endometrial samples can aid in identifying deviations in infertile patients. A similar strategy has been used with great success in cancer research. However, large collaborative initiatives, international uniform protocols of sample collection and processing are crucial to ensure reliability and reproducibility. Overall, the proposed approach holds promise for an objective and accurate classification of endometrial-based infertility and has the potential to improve diagnosis and treatment outcomes.

## Introduction

Infertility is a complex and multifactorial condition that affects millions of couples worldwide (Boivin *et al.*, 2007; Mascarenhas *et al.*, 2012). To date, despite advances in ARTs, success rates of fertility treatments have not substantially improved (Andersen *et al.*, 2005; Smith *et al.*, 2015; Kushnir *et al.*, 2017) and the underlying causes of infertility remain unknown in up to 30% of the infertile couples (Gunn and Bates, 2016). The classification of infertility is currently based on clinical, subjective criteria that are the resultant of consensus (Zegers-Hochschild *et al.*, 2017). However, this classification is insufficient because it does not take into account the potential molecular and cellular changes that may occur during a couple’s process of becoming infertile. Thanks to advances in sequencing technology and single-cell analysis (Li and Wang, 2021) we can now have a deeper understanding of the molecular and cellular changes in infertility.

Although it has long been proposed that the endometrium may play a key role in the development of infertility (Santamaria and Simón, 2021), its exact contribution is not sufficiently clear. Previous studies have shown that disruptions in certain signaling pathways may be responsible for the development of endometrial dysfunction, potentially leading to infertility (Nakamura *et al.*, 2006; Grewal *et al.*, 2008; Aghajanova *et al.*, 2009; Crosby *et al.*, 2020). Researchers have attempted to link endometrial gene expression profiles, by microarray hybridization or RNA sequencing (RNA-seq), to endometrial receptivity (Kao *et al.*, 2002; Riesewijk *et al.*, 2003; Horcajadas *et al.*, 2004; Haouzi *et al.*, 2009; Díaz-Gimeno *et al.*, 2011; Evans *et al.*, 2012; Chan *et al.*, 2013; Hu *et al.*, 2014; Altmäe *et al.*, 2017), or potentially impaired endometrial receptivity, such as may be the case in unexplained infertility (Altmäe *et al.*, 2010) and recurrent implantation failure (Koler *et al.*, 2009; Tapia-Pizarro *et al.*, 2014; Choi *et al.*, 2016; Koot *et al.*, 2016; Shi *et al.*, 2018; Bastu *et al.*, 2019; Chen *et al.*, 2019, 2021). While these studies have revealed hundreds of significantly up- and downregulated genes in receptive and non-receptive endometrium, there is strikingly little overlap of markers between studies, as demonstrated in Table 1. This complicates the study of relevant downstream pathways that help us understand the role of the endometrium in reproductive failure or success. The heterogeneity between studies is ascribed to differences in study methodologies, such as timing and method of endometrial sampling, selection criteria of the study population, transcriptome profiling methods, genome annotation versions used, pipelines for data processing, and the absence of consistent standards for data presentation (Altmäe *et al.*, 2017) (Tables 1 and 2). Currently, the use of endometrial receptivity markers in clinical practice is still controversial, as the evidence for improving clinical outcome is insufficient (Liu *et al.*, 2022). A crucial problem arises from linking often very detailed sequencing information to definitions of fertility and infertility that, as mentioned, are formulated too generally.

**Table 1.** Overview of top 10 most significantly up- and downregulated genes among endometrial transcriptome studies comparing reproductive failure and success groups.

Study	Comparison and sample sizes	Top 10 most significantly upregulated genes	Top 10 most significantly downregulated genes
Koler *et al.* (2009)	RIF (n = 4) vs fertile (n = 8)	Not reported	Not reported
Lédée *et al.* (2011)	RIF (n = 25) vs fertile (n = 15)	SC4MOL, LOC253264, HTR6, SYNGAP1, LRRC37A3, PICK1, IGKV1D-13, DTNB, EPR, PADI1	ECGF1, A2BP1, NKD2, B4GALNT3, BCL2L10, BCBN6, FBXO10, CYP3A4, TMPRSS2, UNC5CL
Tapia-Pizarro *et al.* (2014)	RIF (n = 5) vs control (n = 6) vs fertile (n = 6)	**RIF vs control:** THRAP3, MYH10, SMCHD1, IL6ST, USP53, BAG4, GPATCH4, KIAA1731, AASDHPPT, PHTF2 **RIF vs fertile:** SYTL3, MAP4, SECISBP2L, CCAR1, SCN2A, TGS1, HP1BP3, KIF21A, LIFR, MYH10	**RIF vs control:** NID1, UNC93B1, CNPY3, SCNN1G, ARF3, SCNN1B, TSPAN1, HN1L, GADD45B, MGST1 **RIF vs fertile:** CLU, UCP2, AGPAT2, ADIPOR1, LIMK2, KIAA0317, NID1, RRM1, CNPY3, RBPMS
Choi *et al.* (2016)	RIF (n = 15) vs control (n = 7)	CSDC2, FOSB, PF4, KRT34, CDH15, FAM153B, ANXA8L, MS4A8, PI15, KLRB1	S100P, PLA2G10, SNTG2, CXCL13, TRPM8, RBP4, LYPD3, C8B, SIX1, RASD1
Koot *et al.* (2016)	RIF (n = 43) vs control (n = 72)	Not reported	Not reported
Pathare *et al.* (2017)	RIF (n = 10) vs control (n = 8)	MSX2, PINX1, SFTA2, GLYCTK, ADIPOR2, LOC645381, ATP6V1G1, ARMCX4, ATG16L1, NBEAL2	GPX3, PAEP, AOX1, CXCL14, GAST, MUC16, DARC, TSPAN8, SLC1A1, KIAA1199
Guo *et al.* (2018)	RIF (n = 22) vs control (n = 18)	Not reported	Not reported
Shi *et al.* (2018)	RIF (n = 7) vs control (n = 5)	CAPN6, PKHD1L1, MEGF10, THEM4, CCL21, SERPINB9, TRPM6, GP2, ATP12A(DZ), MAP2K6	C9orf71, B4GALNT2, MBOAT4, NRAP, ABP1, DPP4, LBP, IGFBP1, GPR110, HAPB2
Bastu *et al.* (2019)	RIF (n = 24) vs fertile (n = 24)	MARK2, TOP1, RBM47, ACTB, MOBIA, NCOA4, ROCK2, SMG5, KIAA1429, MLL5	EEF1A1, C1orf229, XLOC_l2_015397, LOC100130557, SCARF2, SNORD12C, OSTCP2, PCSK1N, TRIM46, RAPGEF6
Chen *et al.* (2019)	RIF (n = 3) vs control (n = 3)	HLA-DRB5, HBG2, NFKBIL1, AMER2, ODF3LI, CRHBP, IFITM4P, NR4A1, AHSP, MADCAM1	PPP1R11, HLA-DRB3, HFE2, RGPD1, PSG4, KIR3DS1, OLFM4, SERBINB3, TM4SF4, FAM133A
Xu *et al.* (2019)	RIF (n = 5) vs control (n = 5)	Not reported	Not reported
Zhou *et al.* (2019)	RIF (n = 27) vs control (n = 27)	Not reported	Not reported
Pathare and Hinduja (2022) (reanalysis of Pathare *et al.*, 2017)	RIF (n = 5) vs control (n = 5)	PIR, UCA1, BTAF1, FLJ21687, LOC100133866, CMTM8, ENPP4, SPAG4, PI15, FZD6	PAEP, GPX3, HABP2, AOX1, TSPAN8, THBD, PPARGC1A, KIR2DL1, AGR2, MUC16
Altmäe *et al.* (2010)	Infertile (n = 4) vs fertile (n = 5)	SCGB3A1, SAA1, ATP1A2, IGH@, TRH, ITGA8, TMEM130, C20orf114, AY172962, OSTbeta	RNU12, ENST00000338834, LOC340073, LOC646241, AF083130, ENST00000350040, C11orf69, LOC727933, GPR78, NCKAP1L
Crosby *et al.* (2020)	Unexplained infertile women: pregnant (n = 9) vs not pregnant (n = 11)	DGKB, BRINP1, IGF1, SHISA6, NTNG1, GLYATL2, NELL2, SLC12A2, ZNF816, ETFBKMT	AMZ1, MMP1, SERPINB3, KCNK3, MATN3, C11orf96, LUM, IL1B, NKAIN1, RASGRP2

RIF, recurrent implantation failure.

**Table 2.** Overview of study methodologies and number of differentially expressed genes among endometrial transcriptome studies comparing reproductive failure and success groups.

Study	Biopsy method	Timing of biopsy	Preservation of tissue	Technology	Number of differentially expressed genes
Koler *et al.* (2009)	Pipelle	Cycle day 21	Stored at −80°C	Microarray hybridization	313 Upregulated: 25 Downregulated: 288
Lédée *et al.* (2011)	Pipelle	7–9 days after ovulation during a monitored natural cycle	RNAlater and stored at −80°C	Microarray hybridization	4519 Upregulated: not reported Downregulated: not reported
Tapia-Pizarro *et al.* (2014)	Not reported	On the 20th day of an artificial cycle (i.e. on the seventh day of progesterone administration)	Snap-frozen and stored at −80°C	Microarray hybridization	965 (RIF vs Control) Upregulated: 218 Downregulated: 747
					1040 (RIF vs Fertile) Upregulated: 884 Downregulated: 156
Choi *et al.* (2016)	Pipelle	Cycle day 21	Snap-frozen at −80°C	Microarray hybridization	641 Upregulated: 164 Downregulated: 477
Koot *et al.* (2016)	Endobiops	LH + 5 to LH + 8	Snap-frozen and stored at −80°C	Microarray hybridization	303 Upregulated: not specified, but estimated ∼58 Downregulated: specified as 81% of genes with decreased expression, thus ∼245
Pathare *et al.* (2017)	Endometrial biopsy curette (Probet, Catalogue No. 4164, Gynétics)	6–7 days after hCG during a long protocol of ovarian	Tissue samples were washed with PBS, homogenized with LN2, dissolved in TRIzol (Invitrogen) and stored at −80°C	Microarray hybridization	937 Upregulated: 519 Downregulated: 418
Guo *et al.* (2018)	Pipe suction curettage (Wallace)	LH + 7	Not reported	Microarray hybridization	2519 Upregulated: 1809 Downregulated: 710
Shi *et al.* (2018)	Dilation and curettage during hysteroscopy	5–7 days after the ovulation	Frozen at −80°C	Microarray hybridization	357 Upregulated: 253 Downregulated: 104
Bastu *et al.* (2019)	Pipelle	LH + 7 to LH + 10 between 12:00 and 15:00	RNAlater and stored at −80°C	Microarray hybridization	641 Upregulated: not reported Downregulated: not reported
Chen *et al.* (2019)	Endometrial aspiration biopsy using a disposable uterine sampler	LH + 3 to LH + 7	Snap-frozen after being immersed in RNA Stabilization Reagent	RNA-sequencing	460 Upregulated: 199 Downregulated: 261
Xu *et al.* (2019)	Pipe suction curettage or hysteroscopy	LH + 7	Immediately stored at −80°C	RNA-sequencing	1508 Upregulated: 987 Downregulated: 521
Zhou *et al.* (2019)	Pipe suction curettage	5–7 days after ovulation	Not reported	RNA-sequencing	2140 Upregulated: 966 Downregulated: 1174
Pathare and Hinduja (2022) (reanalysis of Pathare *et al.*, 2017)	See Pathare *et al.* (2017)	See Pathare *et al.* (2017)	See Pathare *et al.* (2017)	See Pathare *et al.* (2017)	719 Upregulated: 414 Downregulated: 305
Altmäe *et al.* (2010)	Pipelle	LH + 7	Not reported	Microarray hybridization	260 Upregulated: 145 Downregulated: 115
Crosby *et al.* (2020)	Pipelle	LH + 7	RNAlater and stored at −80°C	RNA-sequencing	204 Upregulated: 38 Downregulated: 166

N_2_, liquid nitrogen; PBS, phosphate buffered saline.

We argue, that by sequencing the endometrial transcriptome of healthy and infertile women at single-cell level, it should be possible to consistently identify molecular and cellular signatures that can be used to classify endometrial-based infertility. We propose to establish endometrial gene expression profiles to first reach consensus on physiological patterns as determined in endometrial samples. Then, these new patterns can be used to study infertile patients whether and where they deviate from these basic patterns. Such an approach can contribute to a much greater understanding of the endometrial processes potentially underlying infertility. It may also lead to a more objective and accurate classification of infertility, which can ultimately greatly individualize and improve diagnosis and treatment. This approach also offers a much better possibility to compare results from different research groups in detail. Though the focus here is on transcriptomics analysis, the approach can be adapted for any type of molecular analysis.

## Definition of fertile and infertile

Although there are varying perspectives on defining healthy, fertile women, we propose identification based on standard anatomical and endocrinologic criteria: a normal uterus in medical imaging, regular menstrual cycles and a history of a previous pregnancy established within a year resulting in a full-term delivery. Regarding the selection criteria for the group of infertile women, we recommend the inclusion of women with unexplained infertility and recurrent implantation failure in order to increase the chance of identifying an endometrial factor. While the application of these criteria may still encompass the inclusion of women with a variety of genetic profiles, we expect that our proposed method will uncover distinct patterns of endometrial function. Moving forward, groups are categorized based on molecular patterns, extending the analysis beyond clinical phenotypes to enable deeper exploration of underlying biological mechanisms.

## Sample collection

To ensure the reliability and reproducibility of such an approach, an international standardization on how to collect and process samples is essential. This would involve developing a standardized protocol for the collection, storage, and processing of endometrial tissue and single cells to ensure consistency across different studies and laboratories. While logistical constraints may limit full standardization, this does not necessarily impede analysis. For example, when considering biopsy planning, natural cycle biopsies offer a physiologically accurate endometrial representation, while tissue obtained during artificial cycles allows rapid biobanking, despite potential gene expression alterations from exogenous hormones. As our approach involves clustering biopsies by gene profiles, the focus lies on exploring underlying biological mechanisms. Thus, regardless of the biopsy source, those with similar gene profiles will group together. This facilitates the identification of common pathways and the formulation of hypotheses that may play a role in endometrial-based infertility. Finally, linking clinical data to these biopsies can help in shaping these hypotheses.

## A large single-cell transcriptome database

While several databases of endometrial single-cell transcriptomes already exist (Simon, 2020; Wang *et al.*, 2020; Garcia-Alonso *et al.*, 2021), sample sizes are relatively small. Large, geographically broad collaborative initiatives should be encouraged, allowing rapid and large-scale biobanking of endometrial tissue within a consortium with uniform protocols and standardized methods, more clinical, molecular and bioinformatical expertise and better generalizability of the data, altogether improving the quality of the research. Single-cell sequencing allows for the dissection of gene profiles in individual cells, enabling the analysis of patterns for each cell type in each cycle stage and accounting for the substantial variation in gene expression within and between menstrual cycles. Incorporation of single-cell high-throughput data from *in vitro* models that recapitulate *in vivo* conditions, such as the organoid models, could provide valuable data by disease modeling. In addition, endometrial cells that are obtained in a noninvasive way, such as via menstrual blood, are an alternative to the more invasive endometrial biopsy (Shih *et al.*, 2022), and could facilitate large-scale banking of endometrial tissue and data. This would simplify the inclusion of tissue from healthy donors not receiving clinical care, albeit limited to the menstrual phase. Additional sources could involve women undergoing preimplantation genetic testing cycles or donor insemination, predominantly comprising women with a male factor infertility, single women and lesbian couples, in whom an endometrial factor is likely absent.

A similar strategy has been used with great success in cancer research. The TCGA (Cancer Genome Atlas) project, started in 2005, generated comprehensive molecular profiles of different types of cancer (The Cancer Genome Atlas, 2005; Tomczak *et al.*, 2015). Molecular profiling has transformed cancer treatment by guiding the selection of targeted therapies, identifying biomarkers, understanding resistance mechanisms, guiding the use of immunotherapies and facilitating clinical trials (Lee *et al.*, 2018; Liu *et al.*, 2023; Sahm *et al.*, 2023; Saito-Adachi *et al.*, 2023). This personalized approach has improved treatment outcomes and reduced unnecessary side effects. Combined with recently highly developed analytics technology including AI-based methods, this approach has opened doors for the development of innovative therapies in the era of precision medicine. A similar approach could be applied to endometrial-based infertility, where a large database of sequencing data from healthy and infertile endometrial tissue at single-cell level could provide new insights into the molecular mechanisms potentially underlying endometrial-based infertility, which may lead to new therapeutic strategies. However, to confirm the practical effectiveness of this approach, the findings of the hypothesis-free work still need to be extrapolated to the clinic. To this end, a future study will need to link the molecular data with clinical data on (in)fertility.

## Challenges in single-cell transcriptomics

Finally, although single-cell transcriptomics studies offer valuable insights into gene expression at a cellular level, they come with certain limitations. Technical noise, data complexity and dropout events can compromise accuracy and analysis. Additionally, defining cell types accurately, addressing batch effects and managing scalability present additional challenges (Hicks *et al.*, 2018; Lähnemann *et al.*, 2020; Adil *et al.*, 2021). Nonetheless, ongoing advancements in technology and computational methods address many of these disadvantages.

## Conclusions

In conclusion, a classification system derived from sequencing data of endometrium tissue of healthy and infertile women at single-cell level can provide a more objective and accurate classification of endometrial-based infertility. However, the standardization of sample collection and processing is crucial to ensure the reliability and reproducibility of such an approach. While full standardization may present challenges, our key message underscores the importance of establishing a large, accessible biobank/database which can aid in identifying specific molecular patterns. Such an approach has been successful in cancer research and could provide new insights into the molecular mechanisms underlying endometrial-based infertility, ultimately leading to improved diagnosis and treatment.

## Authors’ roles

B.B., E.K., and E.N. drafted the manuscript. B.B., A.I.A., E.K., S.A., G.S., S.M., S.F., F.B., and E.N. revised the initial drafts of the manuscript. E.N. and F.B. critically appraised the pre-final manuscript, after which B.B. adjusted the manuscript for submission. All authors approved submission of the final version.
